# Routine investigations for patients with mental and behavioural disturbances

**DOI:** 10.4102/sajpsychiatry.v29i0.2069

**Published:** 2023-08-31

**Authors:** Solomon M.K.K. Jere, Candice Van Koningsbruggen, Mignon Du Toit, Clint Hendrikse

**Affiliations:** 1 Division of Emergency Medicine, Department of Family, Community and Emergency Care, Faculty of Health Sciences, University of Cape Town, Cape Town, South Africa; 2 Department of Family Medicine and Emergency Medicine, Faculty of Health Sciences, Stellenbosch University, Cape Town, South Africa

**Keywords:** emergency medicine, medical clearance, length of stay, crowding, mental and behavioural disturbance

## Abstract

**Background:**

The process of medical clearance aims to exclude a general medical condition as an underlying cause for mental and behavioural disorders and involves routine screening with special investigations. Current evidence, however, suggests that clinician gestalt should guide the need for special investigations and that there is no benefit to routine screening.

**Aim:**

This study aimed to determine the effectiveness of and adherence to the Western Cape (WC) provincial guidelines for routine investigations of adult patients with behavioural disturbances.

**Setting:**

This study was conducted at Mitchells Plain Hospital in Cape Town, South Africa.

**Methods:**

This descriptive study was conducted at Mitchells Plain Hospital in Cape Town, South Africa. Data were collected from existing electronic registries over a 6-month period. Adult mental healthcare users were risk stratified into the probability of having a general medical condition and the results of their special investigations were described against their outcome.

**Results:**

Of the 688 patients included in this study, 66% had abnormal vital signs and of the 312 patients who received special investigations, 56% were abnormal, including 18% who were clinically significantly abnormal. Abnormal special investigations changed the clinical outcome for 3 (<1%) patients. Adherence to the guidelines was reasonable (82%) but non-adherence resulted in unnecessary investigations.

**Conclusion:**

The results of this study support the existing evidence that clinical assessment and clinician gestalt should guide the need for special investigations and that there is no benefit to routine screening in the emergency centre (EC). The results also demonstrate that non-adherence rarely changed patient outcomes.

**Contribution:**

This study provides information on the value of routine screening investigations in ECs.

## Introduction

Mental and behavioural disorders are one of the leading causes of years lived with disability (YLDs) worldwide.^1^ The vast majority of this burden (80%) occurs in low- and middle-income countries (LMICs) where nearly 20% of health-related disabilities are attributable to mood, psychotic and substance abuse disorders.^2^ In South Africa, the 12-month prevalence of mental health disorders is 16.5%, with an estimated lifetime prevalence of 30.3%.^3^ The reported prevalence in the Western Cape province is the highest nationally with a 12-month and lifetime prevalence of 39.4%.^3,4^ Despite this significant burden, mental and behavioural disorders continue to be a low priority with regard to resource allocation.^5,6,7^

The process of medical clearance aims to exclude a general medical condition (GMC) as an underlying cause for the mental and behavioural disorders and involves routine screening with special investigations.^8,9,8,10^ Previously (1980), this process was reported to add significant value with 34% to 46% of patients with mental and behavioural disorders found to have underlying medical conditions.^11^ However, more recent evidence suggests that appropriate special investigations should rather be guided by clinical history, vital signs and physical examination and not performed routinely.^12,13,14^ Patients with a primary mental and behavioural complaint with normal vital signs and a normal physical exam have less than 1% chance of having a clinically significant abnormal special investigation.^15,16^ A local study conducted at a district-level hospital by Credé et al. demonstrated that routine laboratory screening provides no additional information in the assessment of patients with mental and behavioural disturbances on presentation at the emergency centre (EC).^8,9,17,18^

Zwank et al. performed a before-and-after study at a tertiary hospital where an institutional policy to eliminate routine special investigations for all patients with mental and behavioural disorders was implemented.^19^ Special investigations were subsequently performed at the discretion of the treating clinician, which drastically reduced EC length of stay by a mean of 5 h 30 min, reduced total hospital length of stay by 16 h and decreased the EC laboratory costs per patient by 78%. More importantly, there was no significant increase in adverse events or number of medical referrals and no deaths were reported.^19^ This approach is supported by local and international evidence, as well as professional bodies such as the American College of Emergency Physicians (ACEP) and American Psychiatric Association (APA).^13,17,20,21,22,23,24^

Routine screening comes at a cost, adding to the financial burden, staffing requirements, risk of needle stick injuries and patient discomfort, as well as contributing significantly to EC crowding and staff and patient safety. ‘Crowding exists when there is no space left to meet the timely needs of the next patient requiring emergency care’.^25^ Crowding in the EC negatively affects quality of care, increases medical error and increases morbidity and mortality of all patients in the EC.^25,26^ With prolonged EC length of stay, the risk to patient and staff safety also increases, as patients with mental and behavioural disturbances are often difficult to contain in a chaotic EC environment. The risk–benefit ratio between appropriate screening practices and the risks associated with EC crowding and staff safety should always be considered.

Mitchells Plain District Hospital’s EC follows the Western Cape provincial ‘guideline for routine investigations for exclusion of GMCs in patients presenting with mental and behavioural disturbances’.^27^ The guideline is aimed at reducing length of stay and categorises patients into three groups: (1) patients at high risk for a GMC; (2) known mental healthcare users (MHCU) who are not at high risk for a GMC; and (3) index MHCUs who are not at high risk for a GMC, based on their clinical features. According to the guideline, special investigations are then performed according to the patient’s risk-category. Index MHCUs require a predefined set of special investigations, and MHCUs at high risk of a GMC require a predefined set of investigations as well as additional tests that are clinically indicated.^17,27^ Currently, there are no data that describe adherence to and effectiveness of the given guidelines. The data from Credé et al. was sourced more than 10 years ago and the mental health disease burden and community profiles have changed significantly since then.^4^ The aim of this study was therefore to assess the adherence to and effectiveness of the Western Cape provincial guideline for routine investigations of adult patients with mental and behavioural disturbances presenting to a district-level EC.

## Research methods and design

### Study design

This study is a descriptive analysis and data were collected retrospectively from existing databases and registeries.

### Study setting

This study was conducted at Mitchells Plain Hospital (MPH) in Cape Town, South Africa, which is in the Mitchells Plain Health District of the Western Cape province Metro Region. The hospital is 32 km from Cape Town’s central business district and provides services to an estimated population of 600 000.^28^ It serves low-to middle-income communities of Mitchells Plain and mainly low-income communities of Philippi: A large nearby informal settlement. The MPH is a district-level hospital that has a psychiatric department that is headed by a single psychiatrist and has both a male and female ward on site.

### Study population and sampling

#### Inclusion criteria

All adult patients (18 years and older) with mental and behavioural disturbances who presented to Mitchells Plain Hospital’s EC between July 2019 and December 2019 were eligible for inclusion. For the purpose of this study, the definition of ‘mental and behavioural disturbances’ included any diagnosis from the World Health Organization’s (WHO) international classification of diseases (ICD-10) Version: 2019 Chapter V: Mental and Behavioural Disorders (F00–F99).

#### Exclusion criteria

Patients who were admitted directly to the psychiatric department and bypassed the EC were excluded as well as those who were discharged directly from the EC, transferred to a different hospital, or directly admitted to another discipline. Patients who were clinically delirious with a clear cause (GMC) and referred to the appropriate discipline were not included in this study as the guideline does not apply to them.

### Data collection and management

Data were collected in three phases according to the principles outlined by Gilbert et al. and Lowenstein et al. to minimise inconsistencies.^29,30^ In Phase I, a retrospective review of the data of all included patients was performed. Data were exported from the electronic registry, Hospital and Emergency Centre Tracking and Information System (HECTIS) along with patients’ demographic details, their disposition category, folder numbers and initial vital signs on triage. An ICD-10 search from within the database was carried out by the database manager and de-identified data were exported to a spreadsheet. Folder numbers were used to track patients through all the phases of data collection. Variables from HECTIS, like the vital signs, triage information and process times are entered in real time and crosschecked by different categories of users (clinicians, clerks, nurses, etc.), which ensures accurate and reliable data.

Clinical records of patients identified from Phase I were accessed from the electronic medical records (Enterprise Content Management [ECM]) during Phase II. The ECM is an official electronic database for the Western Cape Health Department where all clinical notes are stored electronically. The notes were scrutinised, and patients were grouped into three categories according to their risk of GMC as a cause for their symptoms of mental and behavioural disturbance as per the Western Cape (WC) guideline. The South African Triage Scale (SATS) was used to group patients into triage categories (colour coded as green, yellow, orange and red) based on their presenting complaints, vital signs, clinical discriminators and severity of illness on presentation to the EC.^31^ An adult MHCU who is aggressive on presentation is triaged orange according to SATS, indicating that such a patient needs very urgent care.^31^ In this study, the following thresholds were used for normal vital signs: systolic blood pressure: 90 mmHg – 139 mmHg, diastolic blood pressure: 60 mmHg – 89 mmHg, heart rate: 60 bpm to 100 bpm, respiratory rate: 12 bpm – 20 bpm, oxygen saturation: 94% – 100% and temperature: 35°C – 37.5°C.^32^ The electronic records were primarily reviewed by two investigators, and a third investigator randomly cross checked ~5% of the case files to ensure that data collection was accurate.

In Phase III of the data collection process, the results of all special investigations performed on admission by the EC were obtained from the National Health Laboratory Services (NHLS) database and were classified into normal, clinically not significantly abnormal, or clinically significantly abnormal. This was also performed for results of special investigations carried out within 48 h of admission by the psychiatric department to ensure that patients with clinically significantly abnormal results, who were not otherwise investigated by EC, were not missed. Clinically significantly abnormal values were based on evidence and international consensus and defined thresholds for what is considered likely to contribute towards psychotic symptoms.^13,17,33,34,35^ Table 1 summarises the reference values that were used.

**TABLE 1:** Reference values for laboratory investigations.

Laboratory investigation	Values considered abnormal enough to contribute towards psychotic symptoms
Sodium	< 125 or > 160 mmol/L
Creatinine	> 200 μmol/L
White cell count	< 4 or > 15 × 10^9^/L
Lumbar puncture	Any polymorphs/µL
	> 3 lymphocytes/µL
	Protein > 0.45 g/L
	Positive gram stain and/or India ink stain and/or syphilis serology positive
HIV rapid test	Positive
Syphilis serology TPHA	Positive
Thyroid-stimulating hormone	< 0.27 or > 4.20 mIU/L

*Source*: Adopted from Credé A, Geduld H, Wallis L. Assessment of routine laboratory screening of adult psychiatric patients presenting to an emergency centre in Cape Town. S Afr Med J. 2011;101(12):891–894^17^

TPHA, rapid plasma reagin

Results of special investigations performed by both EC and the psychiatric department were assessed to determine whether they changed patients’ outcomes. A change in outcome of patients was defined as the need for patients to be transferred to the medical or surgical department from the psychiatric ward. In addition, whether or not psychiatric inpatients received medical consultations (co-management) as a psychiatric inpatient, was also documented.

The effectiveness of the guideline was defined as how well it could distinguish between patients with mental and behavioural disorders as a result of a GMC and MHCU requiring psychiatric admission. To determine adherence, the proportion of patients who had special investigations according to the guidelines was calculated and expressed as a percentage per risk category.

### Data analysis

All variables were described with summary statistics. Categorical variables were described as proportions or percentages and tabulated as necessary. The central tendency of continuous variables was described as medians, and quartiles were used to indicate spread. Tabulated variables were expressed as row percentages when outcome variables are grouped in columns and column percentages were used when descriptive variables are grouped in columns. Data were analysed using Statistical Package for Social Sciences (SPSS) Statistics Version 27.0 (IBM Corp. Released 2019; Armonk, NY: IBM Corp.).

### Ethical considerations

Even though this study involved a vulnerable population, it posed a very low risk to patients as data were de-identified to protect patient identities. The data collection procedures only involved a folder and database review and no patient intervention or interaction occurred. Ethical approval was granted by the Human Research Ethics Committee of the University of Cape Town: HREC REF:678/2020 and institutional approval was obtained from MPH via the National Health Research Database: WC_202012_014.

## Results

**FIGURE 1:**
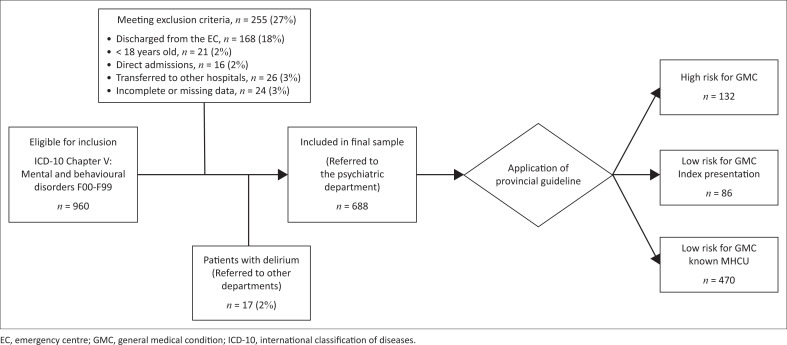
Flowchart of study participants.

A total of 19,162 adult patients presented to MPH EC during the study period of which 960 (5%) were eligible for inclusion into the study. A sample of 688 (72%) were included, and Figure 1 provides a breakdown of the 272 (28%) exclusions. Among the excluded patients are 17 (2%) who had a clear diagnosis of a delirium for various reasons and were subsequently referred to relevant inpatient departments for admission.

Table 2 provides demographical and clinical details of the sample for each admission category. The sample had a strong male preponderance 455 (66%) and nearly 551 (80%) of all patients were younger than 45 years old. Based on their clinical details, 470 (68%) were known mental health users with no features of a GMC, 86 (13%) were index MHCU with no high-risk features of a GMC, and 132 (19%) had clinical features that increased their risk of having a GMC. The ratio of 2:1 male predominance extended to the categories where no high-risk features for GMC were present, while nearly 50% (60 out of 132) of all MHCUs with high-risk features of a GMC were female. The highest proportion of MHCUs at high risk of a GMC and index presenters were in the 18–25-year-old category while the highest proportion of known MHCUs were in the 26–35-year-old category.

**TABLE 2:** Demographical and clinical details for each admission category: *n* (column%).

Characteristic	Total *n* = 688				Low risk for a GMC								High risk for a GMC *n* = 132 (19%)			
	*n*	%	Median	IQR	Known MHCU *n* = 470 (68%)				Index presentation *n* = 86 (13%)				*n*	%	Median	IQR
					*n*	%	Median	IQR	*n*	%	Median	IQR				
**Gender**																
Male	455	66	-	-	316	67	-	-	67	78	-	-	72	54	-	-
Female	233	34	-	-	154	33	-	-	19	22	-	-	60	46	-	-
**Age categories**																
18–25	151	22	-	-	71	15	-	-	35	41	-	-	45	34	-	-
26–35	237	34	-	-	175	37	-	-	27	31	-	-	35	27	-	-
36–45	163	24	-	-	124	26	-	-	15	17	-	-	24	18	-	-
46–55	74	11	-	-	52	11	-	-	6	7	-	-	16	12	-	-
56–65	45	7	-	-	33	7	-	-	3	4	-	-	9	7	-	-
> 65	18	3	-	-	15	3	-	-	0	0	-	-	3	2	-	-
Triage category*																
Green	2	0.3	-	-	2	0.4	-	-	0	0	-	-	0	0	-	-
Yellow	2	0.3	-	-	1	0.2	-	-	0	0	-	-	1	0.8	-	-
Orange	677	99	-	-	464	99	-	-	86	100	-	-	127	96	-	-
Red	6	1	-	-	2	0.4	-	-	0	0	-	-	4	3	-	-
**Diagnosis category**																
F00–F09: Organic, including symptomatic mental disorders	1	0.1	-	-	1	0.2	-	-	0	0	-	-	0	0	-	-
F10–F19: Mental and behavioural disorders because of substance use	50	7	-	-	35	8	-	-	6	7	-	-	9	7	-	-
F20–F29: Schizophrenia, schizotypal and delusional disorders	491	71	-	-	343	73	-	-	62	72	-	-	86	65	-	-
F30–F39: Mood disorders	124	18	-	-	85	18	-	-	15	17	-	-	24	18	-	-
F40–F49: Neurotic, stress-related and somatoform disorders	8	1	-	-	2	0.4	-	-	1	1	-	-	5	4	-	-
T50.9/Z91.5: Intentional self-harm and/or overdose	13	2	-	-	4	1	-	-	2	2	-	-	7	5	-	-
Other	1	0.1	-	-	0	0	-	-	0	0	-	-	1	1	-	-
**Process times (hours: minutes)**																
Time to triage	-	-	0:31	0:13–1:02	-	-	0:31	0:14–1:05	-	-	0:21	0:12–0:57	-	-	0:33	0:13–0:56
Time to consultation	-	-	2:09	0:50–4:41	-	-	2:09	0:49–4:40	-	-	2:13	1:14–5:09	-	-	2:13	0:49–4:23
Time to disposition	-	-	2:39	0:55–8:42	-	-	1:32	0:39–3:35	-	-	8:08	4:11–15:47	-	-	10:52	5:18–17:49
Time to exit	-	-	2:52	0:55–6:31	-	-	3:17	1:18–7:00	-	-	2:13	0.09–6:22	-	-	2:04	0:18–5:16
EC length of stay	-	-	12:19	7:33–20:51	-	-	10:47	6:39–17:12	-	-	17:35	11:34–25:59	-	-	18:54	10:51–25:56

Note: Percentages may not add to 100% because of rounding.

GMC, general medical condition; MHCU, mental healthcare user; IQR, inter quartile range; EC, emergency centre.

* , Triage categories were based on the SATS.^31^

Nearly all (99%) of the patients were triaged as orange, and the majority (68%) were known MHCU. Of those with high-risk features of a GMC, four (3%) were triaged red. MHCU with schizophrenia, schizotypal and delusional disorders contributed the most to the psychiatric burden with 491 (71%) of all admissions. Patients who required special investigations had a longer EC length of stay (7.5 h per patient), contributing to EC crowding – this includes all index patients and those at high risk for GMC (218 patients).

Table 3 depicts all abnormal vital signs for each admission category with subsequent psychiatric department outcomes. A total of 455 (66%) of all the patients included in the final sample had one or more abnormal vital sign. The most prevalent abnormal vital signs were diastolic blood pressure (32%), heart rate (29%) and systolic blood pressure (28%). One patient (0.2% of all patients who had one or more abnormal vital sign) with both an abnormal heart rate and an abnormal, diastolic blood pressure had a change in outcome (transferred to a medical ward), while seven patients (1.5% of all patients who had one or more abnormal vital sign) had medical consultations as a psychiatric inpatient. The distribution of abnormal vital signs showed little variation between the categories with known MHCUs 314/470 (67%), index presenters 52/86 (60%) and those with high-risk features for GMC 89/132 (67%) having one or more vital signs abnormal.

**TABLE 3:** Vital signs on presentation for each emergency centre admission category with subsequent psychiatric department outcomes.

Vital signs	Total		Emergency centre admission category						Psychiatric department outcomes			
	*n*	% of sample	Low risk for a GMC				High risk for a GMC *n* = 132 (19%)		Medical transfer (to medical ward)		Medical consultation (in psychiatric ward)	
			Known MHCU *n* = 470 (68%)		Index presentation *n* = 86 (13%)							
			*n*	Row %	*n*	Row %	*n*	Row %	*n*	Row %	*n*	Row %
All vital signs normal	233	34	156	67	34	15	43	19	2	1	1	0.4
Any abnormal vital sign	455	66	314	69	52	11	89	20	1	0.2	7	2
**Abnormal vital signs**												
Heart rate (per minute)	189	29	132	70	20	11	37	20	1	1	4	2
Systolic blood pressure (mmHg)	180	28	114	63	20	11	46	26	0	0	6	3
Diastolic blood pressure (mmHg)	210	32	149	71	21	10	40	19	1	1	4	2
Oxygen saturation (%)	6	1	5	83	1	17	0	0	0	0	0	0
Respiratory rate (per minute)	39	6	26	67	5	13	8	21	0	0	1	3
Temperature (degrees Celsius)	13	2	6	46	1	8	6	46	0	0	0	0
POC Haemoglobin (g/dL)	79	14*	49	62	8	10	22	28	0	0	3	4
POC Blood glucose level (mmol/L)	33	5	21	64	6	18	6	18	0	0	1	3

Percentages may not add to 100% because of rounding; GMC, general medical condition; POC point of care; MHCU, mental healthcare users.

* , Haemoglobin (g/dL) performed in only 583 (85%) patients.

Table 4 depicts the abnormal special investigations on presentation for each EC admission category with subsequent psychiatric department outcomes. Special investigations were performed in 312 (45%) of the sample, of which 137 (44%) were normal, 56 (18%) clinically significantly abnormal (including rapid human immunodeficiency virus [HIV] test, treponema pallidum haemagglutination Assay [TPHA], rapid plasma reagin [RPR], chest X-ray, computerised tomography[CT] of the brain and lumbar puncture) and 119 (38%) not clinically significantly abnormal. This equates to a total of 175 abnormal results – 25% of the entire sample. Three patients (<1%) had a change in outcome (transferred to the medical department from the psychiatric ward) while five patients (2% of all patients who had special investigations) had medical consultations as a psychiatric inpatient (co-managed). No clinically significantly abnormal sodium or creatinine results were present, and of the 14 clinically significantly abnormal white cell counts and 14 clinically significantly abnormal thyroid stimulating hormone levels, none required transfer to the medical department and was co-managed (by internal medicine and psychiatry) as psychiatric inpatients. The only patient with clinically significantly abnormal results who had a change in outcome was a MHCU with neurosyphilis.

**TABLE 4:** Abnormal special investigations on presentation for each emergency centre admission category with subsequent psychiatric department outcomes.

Special investigations	Total				Emergency centre admission category						Psychiatric department outcomes *n* (row %)			
	*n*	% of Sample	*n*	% of special investigations performed	Low risk for a GMC				High risk for a GMC *n* = 132 (19%)		Medical transfer (to medical ward)		Medical consultation (in psychiatric ward)	
					Known MHCU *n* = 470 (68%)		Index presentation *n* = 86 (13%)							
					*n*	Row %	*n*	Row %	*n*	Row %	*n*	Row %	*n*	Row %
**Sodium (Total)**	268	39	83	31	21	25	26	31	36	43	1	< 1	3	4
(125–134) or (146–160) mmol/L	-	-	83	31	21	25	26	31	36	43	1	< 1	3	4
< 125 or > 160 mmol/L	-	-	0	0	0	0	0	0	0	0	0	0	0	0
**White cell count (Total)**	297	43	71	24	27	38	17	24	27	38	1	< 1	1	< 1
10.4–15 × 10^9^/L	-	-	57	19	21	37	14	25	22	39	1	2	1	2
< 4 or > 15 × 10^9^/L	-	-	14	5	6	43	3	21	5	36	0	0	0	0
**Creatinine (Total)**	299	43	28	9	14	50	5	18	9	32	1	4	1	4
97–200 μmol/L	-	-	28	9	14	50	5	18	9	32	1	4	1	4
> 200 μmol/L	-	-	0	0	0	0	0	0	0	0	0	0	0	0
**TSH (Total)**	135	20	40	30	10	25	12	30	18	45	0	0	1	3
(0.27–0.62) or (3.03–4.2) mIU/L	-	-	26	19	8	31	9	35	9	35	0	0	1	4
< 0.27 or > 4.2 mIU/L	-	-	14	10	2	14	3	21	9	64	0	0	0	0
Rapid HIV test	252	37	11	4	3	27	3	27	5	46	0	0	0	0
TPHA	269	39	20	7	11	55	3	15	6	30	1	5	0	0
RPR	20	3	9	45	4	44	1	11	4	44	1	11	0	0
Chest X-ray	15	2	2	13	2	100	0	0	0	0	0	0	0	0
CT brain	5	0.6	1	20	1	100	0	0	0	0	0	0	0	0
Lumbar puncture	24	3	2	8	0	0	0	0	2	100	0	0	0	0

GMC, general medical condition; TPHA, Treponema pallidum hemagglutination; RPR, rapid plasma reagin (syphilis); TSH, thyroid stimulating hormone; MHCUs, mental healthcare users.

MHCU mental healthcare user; Percentages may not add to 100% because of rounding.

Table 5 depicts the abnormal special investigations performed by the psychiatric department within 48 h of admission for each EC admission category with subsequent psychiatric department outcomes. Special investigations were performed in 146 (21%) of the sample of which 86 (59%) were normal, 27 (18%) clinically significantly abnormal (including rapid HIV, TPHA, RPR, chest X-ray, CT brain and lumbar puncture) and 33 (23%) not clinically significantly abnormal. This equates to a total of 60 abnormal results – 9% of the sample. No patients were transferred to the medical department from the psychiatric ward but seven patients (5% of all patients who had one or more abnormal results), received medical consultations as a psychiatric inpatient. No clinically significantly abnormal sodium results were present but of the clinically significantly abnormal creatinine, white cell count and thyroid stimulating hormone results, none required transfer to the medical department and was co-managed (by internal medicine and psychiatry) as psychiatric inpatients.

**TABLE 5:** Abnormal special investigations performed by the psychiatric department within 48 h of admission for each emergency centre admission category with subsequent psychiatric department outcomes.

Special investigations	Total				Emergency centre admission category						Psychiatric department outcomes			
	*n*	% of Sample	*n*	% of special investigations performed	Low risk for a GMC				High risk for a GMC *n* = 132 (19%)		Medical transfer (to medical ward)		Medical consultation (in psychiatric ward)	
					Known MHCU *n* = 470 (68%)		Index presentation *n* = 86 (13%)							
					*n*	Row %	*n*	Row %	*n*	Row %	*n*	Row %	*n*	Row %
**Sodium (Total)**	73	11	22	30	16	73	1	5	5	23	0	0	1	5
(125–134) or (146–160) mmol/L	-	-	22	30	16	73	1	5	5	23	0	0	1	5
< 125 or > 160 mmol/L	-	-	0	0	0	0	0	0	0	0	0	0	0	0
**White cell count (Total)**	95	14	25	26	19	76	3	12	3	12	0	0	2	8
10.4–15 × 10^9^/L	-	-	18	19	15	83	2	11	1	6	0	0	0	0
< 4 or > 15 × 10^9^/L	-	-	7	1	4	57	1	14	2	29	0	0	2	8
**Creatinine (Total)**	78	11	3	4	3	100	0	0	0	0	0	0	0	0
97–200 μmol/L	-	-	2	0	2	100	0	0	0	0	0	0	0	0
> 200 μmol/L	-	-	1	0	1	100	0	0	0	0	0	0	0	0
**TSH (Total)**	57	8	6	11	5	83	0	0	1	17	0	0	1	17
(0.27–0.62) or (3.03–4.2) mIU/L	-	-	4	0	3	75	0	0	1	25	0	0	1	17
< 0.27 or > 4.2 mIU/L	-	-	2	0	2	100	0	0	0	0	0	0	0	0
Rapid HIV test	58	8	5	9	3	60	1	20	1	20	0	0	1	20
TPHA	56	8	4	7	3	75	1	25	0	0	0	0	0	0
RPR	7	1	2	29	2	100	0	0	0	0	0	0	0	0
Chest X-ray	11	2	3	27	1	33	1	33	1	33	0	0	0	0
CT brain	26	4	8	31	4	50	1	13	3	38	0	0	2	25
Lumbar puncture	2	0.3	0	0	0	0	0	0	0	0	0	0	0	0

GMC, general medical condition; TPHA Treponema pallidum hemagglutination; RPR rapid plasma reagin (Syphilis); TSH Thyroid stimulating hormone; MHCUs, mental healthcare users.

MHCU mental healthcare user; Percentages may not add to 100% because of rounding.

Table 6 depicts a summary of special investigations and adherence to guidelines for each EC admission category and the subsequent psychiatric department outcomes. The EC adhered in 91% (78/86) of the index presenter category, 77% (361/470) of the known mental health user category and 95% (125/132) of the high-risk of GMC category. The overall adherence was 82%. Non-adherence did not affect the outcomes for patients in the index presenter and high risk of GMC categories but resulted in 109 patients receiving unnecessary special investigations (not indicated) in the known mental healthcare user category.

**TABLE 6:** Summary of special investigations and adherence to guidelines for each emergency centre admission category and subsequent psychiatric department outcomes.

Summary of variable	Total				Emergency centre admission category						Psychiatric department outcomes			
	*n*	% of Sample	*n*	% of special investigations performed	Low risk for a GMC				High risk for a GMC *n* = 132 (19%)		Medical transfer (to medical ward)		Medical consultation (in psychiatric ward)	
					Known MHCU *n* = 470 (68%)		Index presentation *n* = 86 (13%)							
					*n*	Row %	*n*	Row %	*n*	Row %	*n*	Row %	*n*	Row %
**Emergency centre**														
No special investigations performed	376	55	-	-	361	96	8	2	7	2	0	0	3	< 1
Special investigations performed	312	46	-	-	109	35	78	25	125	40	3	1	5	2
All special investigations normal	-	-	137	44	53	39	34	25	50	37	0	0	1	< 1
Any abnormal special investigations	-	-	175	56	56	32	44	25	75	43	3	2	4	2
Clinically non-significant	-	-	119	38	35	29	32	27	52	44	2	2	4	3
Clinically significant	-	-	56	18	21	38	12	21	23	41	1	2	0	0
**Psychiatric department**														
No special investigations performed	542	79	-	-	-	-	-	-	-	-	-	-	-	-
Special investigations performed	146	21	-	-	-	-	-	-	-	-	-	-	-	-
All special investigations normal	-	-	86	59	57	66	10	12	19	22	0	0	4	5
Any abnormal special investigations	-	-	60	41	44	73	6	10	10	17	0	0	3	5
Clinically non-significant	-	-	33	22	26	43	2	6	5	15	0	0	0	0
Clinically significant	-	-	27	18	18	67	4	15	5	19	0	0	3	11
**Adherence**	**564**	**82**	**-**	**-**	**361**	**77**	**78**	**91**	**125**	**95**	**3**	**< 1**	**5**	**< 1**
**Non-adherence**	**124**	**18**	**-**	**-**	**109**	**23**	**8**	**9**	**7**	**5**	**0**	**0**	**3**	**< 1**

GMC, general medical condition; MHCUs, mental healthcare users.

The guideline correctly categorised the three patients who were transferred from the psychiatric ward to medical department as high-risk patients. However, the use of the guideline could not change the patients’ management plan in the EC. The three patients who were transferred to the medical department had the following reasons: Patient 1 was a 41-year-old female who had an abnormal TPHA and RPR – the institutional policy is that MHCUs are referred before these results are available as the turnaround time for TPHA results is long; Patient 2 was a 57-year-old female who had a clinically significant Vitamin B12 deficiency (not screened routinely in the EC on admission); and Patient 3 was a 76-year-old male patient whose EC laboratory results were normal; he developed seizures in the psychiatric ward and subsequently had an abnormal CT brain scan.

## Discussion

This study demonstrates an 82% adherence to the provincial guidelines for routine investigations for exclusion of GMCs as a cause for mental and behavioural disturbances. The findings suggest that the application of the guideline did not significantly change patients’ outcomes as decisions were based on clinician gestalt instead of results of special investigations, which is consistent with findings of other studies assessing the use of laboratory investigations for medical clearance in patients with mental and behavioural disturbances.^12,13,14,22^ Even though abnormal vital signs and abnormal special investigations were prevalent, they rarely resulted in a change in outcome as only 3 (<1%) patients, out of 312 patients who received special investigations, had a change in outcome. Although adherence was reasonable, non-adherence resulted in financial waste (>100 patients receiving unnecessary investigations) with no change in patient outcomes.

The high proportion of MHCUs with abnormal vital signs (66%) was surprising, considering the fact that abnormal vital signs could be considered a high-risk criterion to predict a GMC.^32^ Despite its prevalence, abnormal vital signs rarely changed the outcome as only 1 (0.2%) MHCU out of those who had abnormal vital signs, had a change in outcome (medical transfer from psychiatric ward). Variation in vital signs is common and evidence suggests that using groups or combinations of abnormal vital signs, together with clinician gestalt, is superior to one abnormal vital sign in isolation.^36^ The single patient who was transferred to the medical department had an abnormal heart rate and diastolic blood pressure. Further research should aim to assess which abnormal vital signs or combinations of abnormal vital signs have clinical value to predict undesirable outcomes in MHCUs.

Abnormal special investigations were prevalent (56% of patients who received special investigations) and included several clinically significantly abnormal results (18% of patients who received special investigations). Despite the high prevalence, it resulted in only three patients being transferred to the medical department from the psychiatric ward. Of the 56 patients (18%) who had clinically significant abnormal results, only 1 patient was referred to the medical department, despite there being 14 patients with clinically significant abnormal white cell counts and 14 with clinically significant abnormal thyroid stimulating hormone levels. Asymptomatic leucocytosis in patients seeking emergency care is fairly common and often transient; however, when it is coupled with clinical signs of a potential GMC, it has much more value to predict serious pathology.^37^ The fact that these MHCUs were declared medically fit and referred to the psychiatric department despite the abnormal special investigations questions the value of routine screening. The disposition of MHCUs was therefore based on clinician gestalt and not on the results of the investigations, which is a practice supported by an existing body of evidence that demonstrates that testing beyond what is clinically indicated for medical clearance in MHCUs rarely changes clinical care.^12,13,20,38,39^ The transfer of three patients from the psychiatric department to the medical ward did not occur as a result of a failure of clinical assessment or a lack of screening investigations.

Abnormal special investigations often resulted in MHCUs receiving medical consults as a psychiatric inpatient (co-management), which is not surprising, as comorbid conditions are prevalent in MHCUs and many medical conditions can be safely managed while admitted in the psychiatric ward.^40^ This questions the utility and cost-effectiveness of performing screening investigations in the EC as opposed to the psychiatric ward, considering that GMC as a cause for mental and behavioural conditions can be screened for reliably and safely with a thorough clinical assessment and clinician gestalt.^12,22^ From a risk-benefit ratio perspective, patients who wait for special investigations in the EC have a much longer EC length of stay, which increases crowding and affects staff and patient safety. In our sample, none of the index presenters’ special investigations affected their outcome and none required medical transfer, despite the presence of abnormal results; however, each MHCU stayed for nearly 8 h longer in the EC. This makes a strong argument that medical workup that does not affect patient outcomes should ideally be performed in the psychiatric ward, especially if a medical department is available for consultations, to decrease EC crowding and allow patients to get to definitive care as soon as possible.

No previous studies have assessed effectiveness and adherence to the Western Cape guideline for exclusion of GMCs in adult patients presenting with mental and behavioural disturbances. Adherence to the provincial guidelines was reasonable (82%) but non-adherence resulted in financial waste (>100 patients receiving unnecessary investigations) with no change in patient outcomes. Reasons for non-adherence were not explored in this study and the financial impact was not quantified. Additional special investigations performed within the first 48 h as psychiatric inpatients also did not result in change in clinical outcomes, further strengthening the argument against routine screening.

### Limitations

This study sample involved a single facility and only included data over 6 months. With regard to external validity, the authors are of the opinion that results are generalisable, because medical clearance in the public sector is standardised and follows the same provincial guideline. The findings of this study depend on clinical gestalt and therefore need to be contextualised: considering the single centre setting, it is reasonable to expect similar results in other emergency physician run ECs. The study results are particularly valid for facilities with both medical and psychiatric departments on site (in the same facility) but may not be applicable to psychiatric hospitals with no medical department (e.g. specialist psychiatric hospitals). Even though data on other investigations were collected (including drug levels, Vitamin B12, urine analysis, pregnancy tests, HIV viral loads and CD4 counts, creatine kinase, drug tests, etc.), it was not reported on as this project focused on routine investigations only, according to the provincial guideline.

Future studies should prospectively investigate the impact of no routine testing on patient outcomes, EC crowding, hospital expenditure and length of stay. Future studies should also involve multiple facilities and include speciality hospitals.

## Conclusion

The results of this study support the existing evidence that clinician gestalt should guide the need for special investigations and that there is no benefit to routine screening in the EC. The results also demonstrate reasonable adherence to the current guidelines even though this rarely affected patient outcomes. Decisions that did change patient outcomes were based on clinical findings and clinician gestalt, not abnormal special investigations, or vital signs, despite these being prevalent. Guidelines, like these should ideally undergo an impact assessment before they are adopted. A potential benefit for one patient may impact the outcomes of other patients indirectly, especially in ECs where crowding and increased length of stay are linked with poor outcomes. The level of emergency care provision should also be considered as guidelines or clinical decision rules rarely trump clinical gestalt in (academic) settings where emergency medicine is practised. In this setting, clinicians chose clinical gestalt over the guideline on numerous occasions, which probably reflects the effectiveness of the guideline. The argument for routine screening with special investigations to occur in ECs is strongly challenged as the risk of the effects on crowding, as well as staff and patient safety, outweighs the lack of benefit from routine special investigations in addition to a thorough clinical examination and clinician gestalt.
